# Khat induced psychotic disorder: case report

**DOI:** 10.1186/s13011-020-00268-4

**Published:** 2020-04-03

**Authors:** Elias Tesfaye, Wolfgang Krahl, Selamawit Alemayehu

**Affiliations:** 1 Psychiatry Department, Jimma, Ethiopia; 2 External consultant for Center for International Health at the hospital of the Ludwig Maximilians-University (CIHLMU), Munich, Germany

**Keywords:** Khat use, Psychotic disorder, Ethiopia, Management, Khat induced psychosis, Substance related disorder

## Abstract

**Background:**

Khat (*Catha edulis*) is a stimulant leaf khat comes from a tree which grows in countries bordering the Red Sea which are along the east coast of Africa and in west Asia. The psycho- active component within these leaves is cathinone. In Ethiopia, Khat is chewed routinely by users for its euphoric effects and as a recreational drug, and chewing khat has an important role as well in both traditional and religious ceremonies. In this case report, we describe the case of a 33-year-old male patient presented with psychotic symptoms after prolonged and heavy khat chewing.

**Case presentation:**

Findings on psychiatric evaluation encompassing detailed history and mental state examination suggest khat induced psychotic disorder severe in full remission; khat use disorder, severe, in early remission.

**Conclusions:**

This case demonstrates that the use of excess khat above 2 bundles for prolonged duration can manifest with psychotic episodes. A small number of case studies had supported a causal relationship between heavy khat use and psychosis or psychotic symptoms. There have been suicidal attempts and homicidal acts in those who used excess and prolonged use of khat. In our case, the improvement attributed to stopping the khat rather than taking antipsychotics.

Therefore, we recommend an urgent social intervention to change the community norms regarding Khat use through psycho-education in media, institution and in person about the relationship between khat use and mental illness. In addition, we suggest the role of religious institutions on the management of khat use not be overlooked. Finally, this study makes a powerful argument for researchers and policy makers to do clinical study to settle a causal effect relation of khat on mental health.

## Background

Khat (*Catha edulis*) is a stimulant leaf khat comes from a tree which grows in countries bordering the Red Sea which are along the east coast of Africa and in west Asia [1–3].

The main psycho-active component within these leaves is cathinone [3–7]. Several biomedical research findings show that cathinone resembles amphetamine in chemical structure and similarly affects the central and peripheral nervous system as well as behavior [4, 6, 8].

There are about 10 million people commonly use khat in East Africa and countries in the Arabian Peninsula [4]. The use of khat has also been noticed to be popular in western society among immigrants, mostly among Somali, Yemeni and Ethiopian communities [4]. Studies suggest that 80–90% of male adults and 10–60% of female adult populations in East Africa consume khat on a daily basis [4].

This study further show that khat use in Ethiopia is more prevalent in ethnic communities with a tradition of khat use but it is now becoming an every-day drug for the general population [9, 10]. Khat chewed routinely by users for its euphoric effects and as a recreational drug, and chewing khat has an important role as well in both traditional and religious ceremonies [2, 11, 12].

A khat chewing session ceremony may persist for at least 3–7 h [2]. Khat chewing has been reported in few case reports to induce psychotic reactions. These are manic illness with grandiose delusions and a paranoid or schizophreniform psychosis with persecutory delusions associated with mainly auditory hallucinations, fear and anxiety, resembling amphetamine psychosis [3, 7, 13, 14].

There have been suicidal attempts and homicidal acts in those who used excess and prolonged use of khat [3, 8, 11]. Few case studies had reported a causal relationship between heavy khat use and psychosis or psychotic symptoms [4]. These psychological effects of khat have been noticed those with excessive and prolonged use of khat [4, 6]. Symptoms rapidly ceases after khat is withdrawn. It has been shown that khat chewing might exacerbate symptoms in patients with pre-existing psychiatric disorder [13].

Studies associate religiosity is associated with reduced drug consumption and with better indicators for the recovery of patients who are receiving medical treatment for drug addiction [1]. Other evidence shows that implication of religiosity, independently of the religion that is practiced, helps in the recovery from drug addiction and reduces the indices of relapse among patients [15]. Some literature suggest that religiosity can help in the process of drug rehabilitation in the following ways: increases in optimism, the perception of social support, resilience, decreases in levels of stress and anxiety [1, 2, 16].

This case report, describe the case of a 33-year-old patient who presented with psychotic symptoms with no family psychiatric history which arise after prolonged and heavy khat chewing, and discuss the issues raised regarding the psychiatric manifestation, and the treatment implications for the illness. We hope that it will add strength to the previous case report’s findings of link between excess khat use and psychotic symptoms. In addition, it makes a powerful argument for researchers and policy makers to do clinical study to settle a causal effect relation of khat on mental health.

## Main body of text

### Case presentation

This is a 33 years old divorced male who holds a master’s degree in developmental studies and had worked as a lecturer at university and this is his first admission and first visit, the information gathered from the patient himself and his elder brother and the information seems reliable. He came to our hospital to get a “legal certificate regarding his capacity to return to work”. Therefore, we admitted him for observation in order to settle his diagnosis and his capacity to return to work.

According to him he started using khat since 2004; initially he started chewing in small amounts for recreational purposes. However, since 2016 his consumption of khat increased from 2 bundles to 4 bundles, followed by increased amount of time in using and getting the substance, frequent absence from work and conflict with his boss. In addition his khat use led him to have frequent conflict with his wife; which ended by divorce in late 2016. After the divorce, he gradually began to show unusual behaviors like difficulty of falling asleep followed by destroying home materials and tearing off his clothes.

Later, he began to have visual hallucination of wild animals like a lion and a snake on the roof while he chews khat. Occasionally, he started hearing voices which arose from his head and as he reported the voices were similar to that of a machine.

Furthermore, he also reported that sometimes while chewing khat he felt as if he was a detached from oneself and as if he was observing himself from outside. He also sees his double talking to him and giving him comment. He also believes that some external agent changes the position of his household material without his will which usually frightened him. In addition, he also feels that some unknown force opens his head and inserts unknown fluid in his body. As a result of this, his elder brother took him to holy water and he stayed there for about 2 months. During this time he had some improvement. However, after 4 months of sobering, he started to use khat in large amounts after having conflict with his boss at work.

In 2018, his illness worsened to the extent; he attempted to kill himself using a rope in the absence of a depressed mood. Two months after his suicidal attempt his family took him to a monastery for holy water treatment. At his stay at the holy water, religious fathers at the monastery advised him not to chew khat again. Because of this, he accepted to quit khat use and his illness improved in 2 weeks’ time of his sober from khat consumption.

### Current presentation

This is a 33 year’s old divorced male person who had a history of mental illness since 2016 and for it he visited holy water treatment on several occasions. The last time he went to holy water was 9 month ago. At which time the religious fathers insisted him to quit using any psychoactive substance and as a result he gave up chewing khat; he became symptom free within 2 weeks and as the patient stated he is relatively better and symptom free for the past 9 months.

Routine laboratory tests including LFT, RFT and thyroid-stimulating hormones were within normal range. The urine toxicology screen of khat was not done. Neuropsychological evaluation at admission at psychiatric ward showed no psychomotor agitation, mannerism, tics and tremors. The quantity of speech was normal, rate and volume.

In mental status examination: there was no Delusion and hallucination. He had no Suicidal/Homicidal Ideation. He is alert and oriented to place, person but not for date. Fund of knowledge and abstract knowledge was intact. He had good concentration and memory. He had Level 6 insight i.e. (true emotional insight of illness). Social and tested judgment was good.

### Course of the illness and management

He has a history of chewing khat for more than 12 years and has increased consumption since.

2016 (3 years back) after which he started to show signs and symptoms such as Irritability, Difficulty of falling asleep, delusion of influence, hallucination and Suicide attempt. As he reported he has been better and symptom free for the past 09 month within 2 weeks after he quit chewing khat.

We admitted him with the diagnosis of Khat Induced Psychosis, in full remission; Khat use Disorder, severe, in early remission. Because he is symptom free for the past 09 month; We just give him education about khat use and its association with mental illness, the benefit of staying sober, the risk of relapse and how to prevent relapse using mindfulness. Finally, a legal certificate written saying “he is fit to return to work; as long as, he sober from khat use”.

## Discussion and conclusion

Based on DSM-5: substance induced psychotic disorder diagnosed when the involved substance is capable of producing the mental disorder; associated with psychotic symptom during or within 1 month of a substance intoxication or withdrawal of taking a substance [17]. In our case, the patient has prolonged use of khat for more than 12 years and the amount of consumption has increased since 3 years back. This was the time where the signs and symptoms such as irritability, Difficulty of falling asleep, delusion of influence, hallucination and Suicide attempt.

Studies showed that not khat consumption per se but rather early onset and excessive khat chewing related to psychotic symptoms. In most cases a pattern of binge chewing (more than two ‘bundles’ per day) preceded the onset of psychotic symptoms [4, 13]. Furthermore, case studies reported that recent khat use history precede the onset of psychotic symptoms [4]. Similarly, in our case the psychotic symptoms worsen with the increment of khat consumption. Other studies also suggest that the prolonged use of khat to be associated with psychotic symptoms [4, 6]. Similar to the existing evidence our case consumes khat for more than 12 years.

As he reported, he has been better and symptom free for the past 09 month. This occurred about 2 weeks after he quit (chewing) khat use. Literatures show that many episodes of khat-induced psychosis resolve spontaneously within 1–2 days of cessation of use; sometimes symptoms subside within less than 24 h [11]. Despite that most clinicians agree that, even when antipsychotics are given, the improvement attributed to stopping the khat rather than taking antipsychotics [11]. In our case symptoms subside after cessation of khat use.

Khat use implicated in homicide and suicide following consumption of khat use [3, 5, 8, 11]. Similar to the existing evidence our case had a history of suicidal attempt after the increment of khat use. The use of amphetamine-like substances including; khat lined as a cause of psychotic disorder in an otherwise healthy individual, or trigger the onset of schizophrenia in an individual with high vulnerability to the disease [7]. In most of those cases, heavy khat consumption preceded the psychotic episodes [3, 5, 8, 11]. Most khat user patients who experienced a relapse of a psychotic illness were found to be those who had resumed khat consumption following discharge [4].

In our setting there is a need also to have objective measurement of khat to detect and monitor khat use in the urine can institute for those who are on follow-up because of the high impact on the relapse rate for the underlying psychiatric symptoms. This can help for confirmation of a suspected khat-induced state. First, a rapid screen by immunoassay detects amphetamine-related compounds [11]. Then gas chromatography mass spectrometry performed. This cannot detect cathinone directly, but rather a positive result that indicates the presence of norephedrine, a cathinone metabolite [11]. The test will give a positive result for up to about 48 h after consumption of khat. The test is highly sensitive, but not highly specific but still helpful [11].

The impact of khat use is significant socially and functionally as elicited in this case where he had divorce because of disagreement and irritability. Some literature shows that khat consumption has adverse consequences for married life [5]. The reason implicated as to contribute to divorce is because spending money to maintain the habit and wasting time at the khat ceremonies lead to family neglect and, consequently, to divorce reported [5]. In some cases deterioration of sexual activities and estrangement between spouses is also reported [5].

The use of khat has enormous effect on the social, economic and mental health aspect of the society. Specific health care needs of patients with histories of khat use and mental illness, and the commitment to implement and provide culturally appropriate health and social care should be considered in future policy developments [4].

Because of cathinone similarity to amphetamine, there is reason to believe that the effect of khat on health is similar to that of amphetamine. The differing effects on health are mainly due to differences in dosage and mode of application [5, 7]. The fact that WHO has recommended that cathinone be put under international control and it is now included in the list of controlled drugs [5, 7]. Despite this the use of khat is legal in Ethiopia. Khat chewing has been a daily practice in many Ethiopian communities for many generations [5].The practice, with its alleged ill effects, is currently spreading throughout the country [5].

Furthermore, caretakers identified khat consumption as the main cause of relapse among psychiatric patients in Ethiopia [6, 18]. There is a need for creating awareness on the adverse effect of khat on mental illness will be effective intervention to reduce the new case of mental illness due to khat use and also in the reduction relapse rate of psychiatric illness. In addition, Law enforcement with long-term intervention plans is needed for preventive intervention and legal systems.

In addition, social interventions to change the community norms regarding Khat use which is crucial, geared by creating recreations alternatives and opportunities. The need for collaboration with religious institutions and establishing addiction rehabilitation centers should get emphasis. There is also a need for clinical study to determine the cause effect relation between khat and mental illness. In addition, further research on khat-related severe mental disorders has to be undertaken in Ethiopia since it considerably complicates treatment of comorbid psychiatric disorders.

In Conclusion**,** there are more than 10 million people commonly use khat in East Africa and countries in the Arabian Peninsula. Khat chewing reported in research to induce psychotic reactions. There have been suicidal attempts and homicidal acts in those who used excess and prolonged use of khat. In our case, we found a relationship between psychosis with heavy consumption of khat more than 2 bundles and use for a long time where, the improvement attributed to stopping the khat rather than taking antipsychotics.

This calls for an urgent social intervention to change the community norms regarding Khat use through psycho-education in media, institution and in person about the relationship between khat use and mental illness. We recommend establishing an addiction rehabilitation center to reduce khat induced psychotic disorder. In addition, we suggest the role of religious institutions on the management of khat use not be overlooked. Finally, we recommend future researchers to conduct clinical study to find the cause effect relationship between khat use and psychosis.

## Data Availability

“Not applicable”.

## Abbreviations

DSM-5: Diagnostic and Statistical Manual of Mental Disorders, Fifth Edition
LFT: Liver function test
RFT: Renal function test
WHO: World Health Organization
